# Research on High-Precision Motion Planning of Large Multi-Arm Rock Drilling Robot Based on Multi-Strategy Sampling Rapidly Exploring Random Tree*

**DOI:** 10.3390/s25092654

**Published:** 2025-04-22

**Authors:** Qiaoyu Xu, Yansong Lin

**Affiliations:** College of Mechanical and Electrical Engineering, Henan University of Science and Technology, Luoyang 471000, China; lys@stu.haust.edu.cn

**Keywords:** multi-strategy sampling, RRT*, deep reinforcement learning, motion planning, multi-arm

## Abstract

In addressing the optimal motion planning issue for multi-arm rock drilling robots, this paper introduces a high-precision motion planning method based on Multi-Strategy Sampling RRT* (MSS-RRT*). A dual Jacobi iterative inverse solution method, coupled with a forward kinematics error compensation model, is introduced to dynamically correct target positions, improving end-effector positioning accuracy. A multi-strategy sampling mechanism is constructed by integrating DRL position sphere sampling, spatial random sampling, and goal-oriented sampling. This mechanism flexibly applies three sampling methods at different stages of path planning, significantly improving the adaptability and search efficiency of the RRT* algorithm. In particular, DRL position sphere sampling is prioritized during the initial phase, effectively reducing the number of invalid sampling points. For training a three-arm DRL model with the twin delayed deep deterministic policy gradient algorithm (TD3), the Hindsight Experience Replay-Obstacle Arm Transfer (HER-OAT) method is used for data replay. The cylindrical bounding box method effectively prevents collisions between arms. The experimental results show that the proposed method improves motion planning accuracy by 94.15% compared to a single Jacobi iteration. MSS-RRT* can plan a superior path in a shorter duration, with the planning time under optimal path conditions being only 20.71% of that required by Informed-RRT*, and with the path length reduced by 21.58% compared to Quick-RRT* under the same time constraints.

## 1. Introduction

With the continuous advancement of tunnel modernization, the intelligentization and automation of multi-arm rock drilling robots have gradually become key industry trends. Robot arm motion planning is a critical research area in robotics [1]. When a multi-arm rock drilling robot performs drilling operations, it requires the robotic arms to autonomously plan the path to the target position while simultaneously ensuring accurate localization, optimal path planning, fast planning, and safe obstacle avoidance.

Robotic arm motion planning consists of two key components: path planning and trajectory planning, with path planning being central to determining performance. For the path planning of multi-joint robotic arms in 3D space, the RRT (rapidly exploring random tree) algorithm is widely used due to its strong capability in high-dimensional spatial search. The RRT algorithm, proposed by Lavalle S M [2] in 1998, randomly selects sampling points, which often results in inefficient and suboptimal paths.

To address the shortcomings of the RRT algorithm, researchers have proposed various solutions [3,4,5,6]. Kuffner et al. [7] proposed the RRT-Connect algorithm, which generates two trees from the start and end points, respectively, which speeds up the path search speed but does not guarantee the optimality of the paths. Karaman et al. [8] proposed the RRT* algorithm, which optimizes the path by reselecting parent nodes and rewiring to solve the problem of poor asymptotic optimality of RRT, but the search efficiency is low. Gammell et al. [9] proposed the Informed RRT* algorithm, which speeds up the search for the optimal solution by sampling point selection in an ellipsoidal state-space region. However, it still relies on undirected exploration in the beginning of the planning phase. Zhang et al. [10] proposed the IPQ-RRT* Connect algorithm for multi-objective point motion planning of assembly robotic arms. Due to the inherent characteristics of the RRT algorithm, the drawback of random selection of sampling points leading to lower search efficiency remains unsolved.

With the rapid development of deep learning, domestic and international researchers began to apply deep reinforcement learning to the motion planning of robotic arms [11,12,13,14,15]. The DRL model is trained through the interaction between the robotic arm and the environment to learn the optimal motion strategy in different states.

Yang et al. [16] proposed a deep Q-learning framework, incorporating dynamic action evaluation and an object separation reward network to effectively realize robotic arm grasping. Zhao et al. [17] proposed the HM-DDPG method, which utilizes joint-space positive kinematics planning and full retention of experience, combined with bias-free hierarchical memory, to improve the efficiency and accuracy of textile robotic arm path planning. Andrychowicz et al. [18] proposed a new technique called “hindsight experience replay”, which can be combined with arbitrary off-policy reinforcement learning algorithms by utilizing sparse binary rewards for efficient sample learning, avoiding complex reward design. Kim et al. [19] proposed a motion planning algorithm that combines the twin delayed deep deterministic policy gradient with hindsight experience replay to solve the path smoothness problem of a robotic arm in a continuous action space through reinforcement learning. Hao et al. [20] proposed a visual servoing method based on target detection and KAN-BiLSTM trajectory prediction, which effectively predicts the trajectory of robotic arm motion and realizes dynamic planning. Liu et al. [21] proposed a DRL path planning system incorporating a safety verification mechanism, which significantly improves the response speed and safety performance of collaborative robots against dynamic obstacles.

In complex tasks, the performance of model-free DRL applied to path planning alone is more limited, and the current trend is to combine DRL with traditional path planning methods [22,23]. Gao et al. [24] proposed a new incremental training model by pre-training the DRL model in a 2D environment and migrating it to a 3D environment, which effectively improves the robot’s path planning efficiency and generalization ability. Cai et al. [25] proposed a path planning framework integrating deep reinforcement learning and the RRT* algorithm and improved the TD3 algorithm to enhance the performance of robotic arm path planning in 2D and 3D environments. Liu et al. [26] proposed DDPG-RRT, a multi-mechanical arm path planning algorithm incorporating deep reinforcement learning, which improves the traditional RRT algorithm by introducing the dynamic step setting of the DDPG algorithm to achieve collision-free path search with synchronized departure of multi-mechanical arms.

In this study, the DRL model is integrated into the sampling point selection of the RRT* algorithm to form the MSS-RRT* algorithm, which is designed to efficiently plan a short and safe motion path for the robotic arm in a short time.

The main contributions of this paper are as follows:

1. A dual Jacobi iterative inverse solution method based on target correction is proposed, which effectively improves the positioning accuracy of motion planning through the dynamic correction of target position.
2. The MSS-RRT* planning algorithm is proposed, incorporating a multi-strategy sampling mechanism that includes DRL position sphere sampling, spatial random sampling, and goal-oriented sampling. It effectively reduces invalid sampling by dynamically switching sampling strategies at multiple stages. It significantly improves the planning efficiency compared to algorithms such as Informed RRT* and Quick-RRT*.
3. In DRL model training, the HER-OAT experience replay mechanism is employed to convert failure data into successful outcomes by adjusting the target joint values and obstacle arm positions. This approach effectively enhances the performance of DRL position ball sampling during the initial stage of planning.

This paper is organized as follows:

In Section 2, the robotic arm is first modeled using forward kinematics, followed by an analysis of the positioning error relationship between forward and inverse kinematics, and the establishment of the forward solution error model. In Section 3, the dual Jacobi iterative inverse solution algorithm based on target correction is proposed. In Section 4, the multi-strategy sampling mechanism is introduced, the process of building and training the DRL model is explained, and the specific approach for MSS-RRT* motion planning is detailed. In Section 5, the effectiveness of the proposed method is validated through simulations and experiments.

## 2. The Establishment of DH Model and Forward Kinematics Error Model

**Figure 1:**
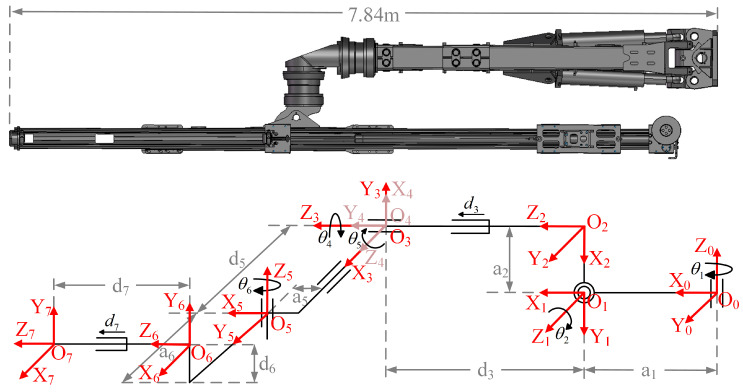
The DH model structure of the robotic arm.

This paper focuses on the G3Zi rock drilling robot, which is equipped with three robotic arms. Each arm consists of five rotary joints and two translational joints, with a total length of approximately 7.84 m, making it a large hydraulic robotic arm. A DH kinematic model is established to calculate the theoretical positions and orientations of the robotic arm’s end effector for various joint configurations. The structure of the robotic arm model is shown in Figure 1.

The DH parameter values for each joint of the model, including the rotation angle $\theta_{i}$, link offset $d_{i}$, twist angle $\alpha_{i}$, and link length $a_{i}$, are presented in Table 1.

**Figure 2:**
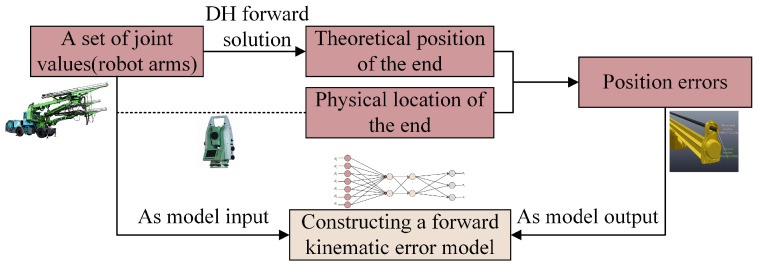
The process of establishing the forward kinematics error model.

The deflection error caused by the huge size of the robot arm itself seriously affects the positioning accuracy of the end of the robot arm. Therefore, it is necessary to build a forward kinematic error model to accurately position the robot arm. The DH method is used to calculate the theoretical position of the end of the robot arm, and the actual position of the end corresponding to the joint value is recorded by the total station. The two positions are subtracted to obtain the end position error. The joint value and the end position error are used as the input and output of the neural network model for training, respectively, to obtain the mapping relationship between the joint value and the position error of the robot arm. The spatial error distance between the actual position and the theoretical position when the robot arm is at different joint angles can be determined. The model building process is shown in Figure 2.

## 3. The Dual Jacobi Iterative Inverse Solution Algorithm Based on Target Correction

In robotic motion planning, solving inverse kinematics is critical. The primary approaches include analytical methods, numerical methods, and intelligent algorithms. The analytical method [27] directly computes joint variables through mathematical derivation, offering real-time performance advantages but being limited by the structural complexity of the manipulator. Intelligent algorithms [28,29] employ data-driven kinematic mapping to handle complex scenarios yet depend on extensive training data and require high computational costs.

The Jacobi iterative method belongs to the numerical solution methods [30]. It provides a general and flexible solution framework for inverse kinematics problems through differential kinematics mapping and iterative approximation, which is especially suitable for multi-DOF robotic arms.

### 3.1. Mathematical Foundations of the Jacobi Iterative Method

The relationship between the generalized velocity vector and the joint velocity vectors is $\dot{x}=J\left(q\right)\dot{q}$, where $J_{ij}\left(q\right)=\partial x_{i}/\partial q_{j}$. Since the robotic arm has seven joints, J(q) is a 6 × 7 matrix. Expand $\dot{x}=J\left(q\right)\dot{q}$ as shown in Equation (1).

(1) $$\left[\begin{array}{c} v_{x}\\ v_{y}\\ v_{z}\\ \omega_{x}\\ \omega_{y}\\ \omega_{z} \end{array}\right]=\left[\begin{array}{ccccccc} J_{11} & J_{12} & J_{13} & J_{14} & J_{15} & J_{16} & J_{17}\\ J_{21} & J_{22} & J_{23} & J_{24} & J_{25} & J_{26} & J_{27}\\ J_{31} & J_{32} & J_{33} & J_{34} & J_{35} & J_{36} & J_{37}\\ J_{41} & J_{42} & J_{43} & J_{44} & J_{45} & J_{46} & J_{47}\\ J_{51} & J_{52} & J_{53} & J_{54} & J_{55} & J_{56} & J_{57}\\ J_{61} & J_{62} & J_{63} & J_{64} & J_{65} & J_{66} & J_{67} \end{array}\right]\left[\begin{array}{c} dq_{1}\\ dq_{2}\\ dq_{3}\\ dq_{4}\\ dq_{5}\\ dq_{6}\\ dq_{7} \end{array}\right]=J_{n}\left[\begin{array}{c} dq_{1}\\ dq_{2}\\ dq_{3}\\ dq_{4}\\ dq_{5}\\ dq_{6}\\ dq_{7} \end{array}\right]$$

In the above equation, $v_{x}v_{y}v_{z}$ represents the end effector’s translational linear velocity, $\omega_{x}\omega_{y}\omega_{z}$ indicates its rotational angular velocity, and $J_{n}$ denotes the arm’s Jacobi matrix.

The relationship between the differential transform in the end coordinate system and the differential transform in the base coordinate system is shown in Equation (2).

(2) dxTdyTdzTδxTδyTδzT=nxnynzp×nxp×nyp×nzoxoyozp×oxp×oyp×ozaxayazp×axp×ayp×az000nxnynz000oxoyoz000axayazdxdydzδxδyδz

Since the rotation axis of the rotary joint is the Z-axis, its differential rotation vector has non-zero components only in the Z-direction, and the corresponding Jacobi matrix column vectors are shown in Equation (3).

(3) $$J_{1,2,4,5,6}={\left[\begin{array}{cccccc} {\left(p\times n\right)}_{z} & {\left(p\times o\right)}_{z} & {\left(p\times a\right)}_{z} & n_{z} & o_{z} & a_{z} \end{array}\right]}^{T}$$

The translation joints move along the Z-axis so that only Z-axis non-zero components exist in their differential translation vectors, and the corresponding Jacobi matrix column vectors are shown in Equation (4).

(4) $$J_{3,7}={\left[\begin{array}{cccccc} n_{z} & o_{z} & a_{z} & 0 & 0 & 0 \end{array}\right]}^{T}$$

### 3.2. Implementation of Dual Jacobi Iteration Algorithm Based on Target Correction

**Figure 3:**
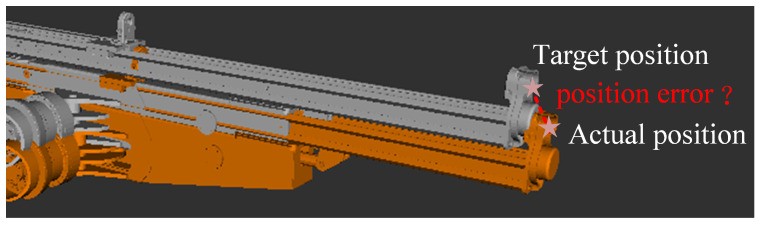
End-effector position error.

The forward solution error compensation model can accurately determine the actual position of the robotic arm’s end, but it does not fully address the positioning deviation between the actual end position and the target end position. This forward kinematic localization error, which is known but not compensated for, becomes the primary factor affecting the accuracy of inverse kinematics. The robotic arm position error is illustrated in Figure 3, where the gray arm represents the target end position, the yellow arm represents the actual position, and the red line segment denotes the end position error.

The dual Jacobi iterative inverse solution algorithm based on target correction effectively solves the above problems. The joint values obtained from the first inverse solution are calculated by DH forward solution and forward solution error compensation model to obtain the end position error. The target position is corrected according to this error, and the final accurate combination of joint values is obtained by the second small-scope Jacobi iteration on the basis of the first inverse solution joint values, which improves the inverse solution localization accuracy.

The symbol reference table for this chapter is shown in Table 2.

**Figure 4:**
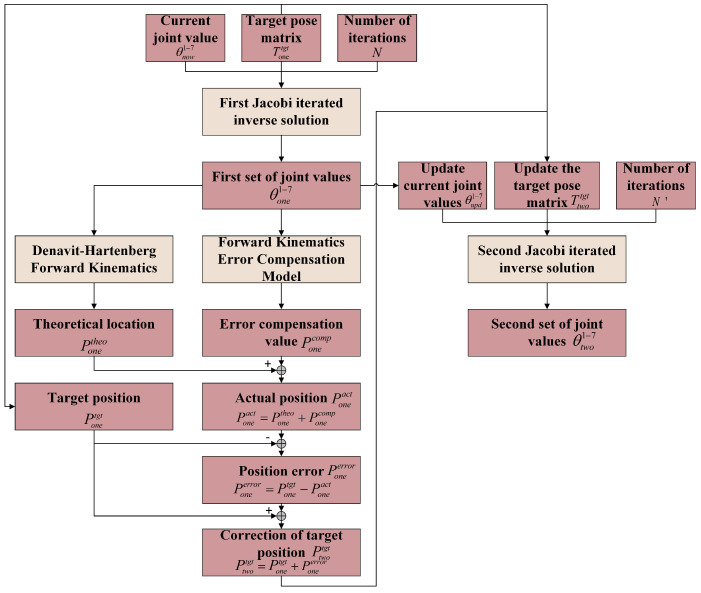
The dual Jacobi iterative inverse solution algorithm based on target correction.

The flowchart of the algorithm is shown in Figure 4.

The specific process is as follows:

1. Firstly, the first Jacobi iteration is performed based on the current joint values $\theta_{now}^{1-7}$, the initial target pose matrix $T_{one}^{tgt}$, and the number of iterations *N* to obtain the first set of inverse solution joint values $\theta_{one}^{1-7}$.
2. Perform DH forward solution on the joint values $\theta_{one}^{1-7}$ to obtain the theoretical position $P_{one}^{theo}$ of the end of the robot arm, and then obtain the end position error compensation amount $P_{one}^{comp}$ through the forward solution error compensation model.
3. $P_{one}^{act}=P_{one}^{theo}+P_{one}^{comp}$: Add $P_{one}^{theo}$ and $P_{one}^{comp}$ to obtain the actual end position $P_{one}^{act}$ of the joint values $\theta_{one}^{1-7}$.
4. $P_{one}^{error}=P_{one}^{tgt}-P_{one}^{act}$: The end position error $P_{one}^{error}$ is obtained by making a difference between the target end position $P_{one}^{tgt}$ and the actual end position $P_{one}^{act}$.
5. $P_{two}^{tgt}=P_{one}^{tgt}+P_{one}^{error}$: The corrected end target position $P_{two}^{tgt}$ (second target position) is obtained by adding the target end position $P_{one}^{tgt}$ to the end position error $P_{one}^{error}$.
6. Enter the second Jacobi iteration and first update the current joint values $\theta_{upd}^{1-7}$. Update the target pose matrix $T_{two}^{tgt}$ according to the corrected end target position, complete the second Jacobi iteration inverse solution, and obtain the exact inverse solution joint values $\theta_{two}^{1-7}$.

## 4. MSS-RRT* Motion Planning Algorithm

By combining deep reinforcement learning with the RRT* algorithm, this approach leverages the intelligent decision-making capability of the DRL model and the powerful search ability of RRT*, offering a novel solution for fast and efficient motion planning.

The middle arm is selected as the main robotic arm, denoted as $A_{main}$, with $A_{main}^{start}$ representing its initial state, $A_{main}^{now}$ indicating the current state, and $A_{main}^{goal}$ denoting the target state. The left and right arms are designated as obstacle robotic arms, denoted as $A_{obs}$.

The symbol reference table for this chapter is shown in Table 3.

### 4.1. Multi-Strategy Sampling Mechanism

The large size of the rock drilling robot arm results in a wide search space. At the same time, the random sampling strategy of the RRT* algorithm makes it difficult to accumulate enough sampling points near the optimal path in a short period of time, which limits the effectiveness of the RRT* algorithm in rewiring and reselecting parent nodes, thus affecting the optimality of the path. In addition, the non-directional sampling strategy of the RRT* algorithm in the initial stage generates a large number of invalid sampling points, which is the key reason for the low efficiency of the algorithm.

Introducing the DRL model to guide the RRT* algorithm for sampling point selection can effectively overcome the disadvantage of lower search efficiency in the initial stage. However, relying on DRL model sampling alone will lose the original advantages of RRT*. In order to solve the above problems, this paper proposes an RRT* algorithm with a multi-strategy sampling mechanism, namely the MSS-RRT* algorithm.

**Figure 5:**
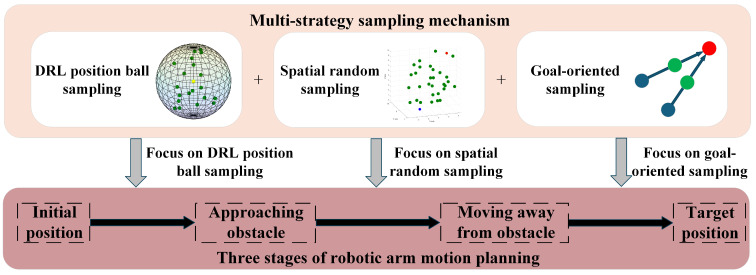
Multi-strategy sampling mechanism.

The MSS-RRT* algorithm is optimized for the sampling strategy of the traditional RRT* with three different sampling methods: DRL position ball sampling (DRL-PBS), spatial random sampling (SRS), and goal-oriented sampling (GOS). The core idea is to dominate DRL position ball sampling in the early planning stage, increase the weight of spatial random sampling when approaching obstacles, and dominate goal-oriented sampling after successfully avoiding obstacles. The principle of the multi-strategy sampling mechanism is shown in Figure 5.

The stage of the $A_{main}^{start}$ approaches the $A_{obs}$, focusing on DRL position ball sampling as it carries directionality, which can avoid the generation of invalid sampling points caused by the wrong starting direction. In the stage when the $A_{main}$ is detached from the $A_{obs}$, the focus is on spatial random sampling, which provides a basic guarantee for the RRT* algorithm to search for the optimal path. In the stage of the $A_{main}$ to the $A_{main}^{goal}$, the target-oriented sampling is the main focus so as to arrive at the target position quickly and accelerate the path search efficiency.

Based on the parameter optimization results from several experiments, this study set the probability of the primary sampling method to 0.8 and the probability of the secondary sampling method to 0.1. The probabilities of the three sampling strategies at the beginning of planning are set to (0.8, 0.1, and 0.1). The critical minimum distance $D_{thresh}$ is set in advance. During the initial to critical states, the collision detection method is used to calculate the minimum distance $D_{now}^{obs}$ between the $A_{main}^{now}$ and the $A_{obs}$ and the minimum distance $D_{start}^{obs}$ between the $A_{main}^{start}$ and the $A_{obs}$, respectively, to update the sizes of $P_{DRL}$ and $P_{SRS}$, as shown in Equation (5). When arriving at the first critical threshold point, the probabilities of the three sampling strategies become (0.1, 0.8, and 0.1), maintained until the second critical threshold point appears. During the process from the second critical threshold point to the target position, $P_{SRS}$ and $P_{GOS}$ are updated by calculating the Euclidean distance $d_{now}^{goal}$ between the end position of the $A_{main}^{now}$ and the end position of the $A_{main}^{goal}$ and combining it with the Euclidean distance $d_{thresh}^{goal}$ between the end position of the $A_{main}$ in the critical state and the end position of the $A_{main}^{goal}$, as shown in Equation (6). When arriving at the $A_{main}^{goal}$, the probabilities of the three sampling strategies become (0.1, 0.1, and 0.8). The multi-strategy sampling mechanism adds constraints to the three sampling strategy probabilities, as shown in Equation (7).

(5) $$\left\{\begin{array}{c} P_{DRL}=0.8-0.7\times \frac{D_{start}^{obs}-D_{now}^{obs}}{D_{start}^{obs}-D_{thresh}}\\ P_{SRS}=0.1+0.7\times \frac{D_{start}^{obs}-D_{now}^{obs}}{D_{start}^{obs}-D_{thresh}}\\ P_{GOS}=0.1 \end{array}\right.$$

(6) $$\left\{\begin{array}{c} P_{DRL}=0.1\\ P_{SRS}=0.8-0.7\times \frac{d_{thresh}^{goal}-d_{now}^{goal}}{d_{thresh}^{goal}}\\ P_{GOS}=0.1+0.7\times \frac{d_{thresh}^{goal}-d_{now}^{goal}}{d_{thresh}^{goal}} \end{array}\right.$$

(7) $$\left\{\begin{array}{c} P_{DRL}+P_{SRS}+P_{GOS}=1\\ 0.1\leq P_{DRL},P_{SRS},P_{GOS}\leq 0.8 \end{array}\right.$$

### 4.2. DRL Model Construction and Training Results

The key components in building a DRL model for a multi-arm rock drilling robot include establishing the foundational framework (such as algorithm selection, defining state and action spaces, and setting the reward function), designing a multi-arm collision detection scheme, and incorporating HER-OAT, an experience replay mechanism that enhances training efficiency.

#### 4.2.1. DRL Model Foundation Framework Construction

**Figure 6:**
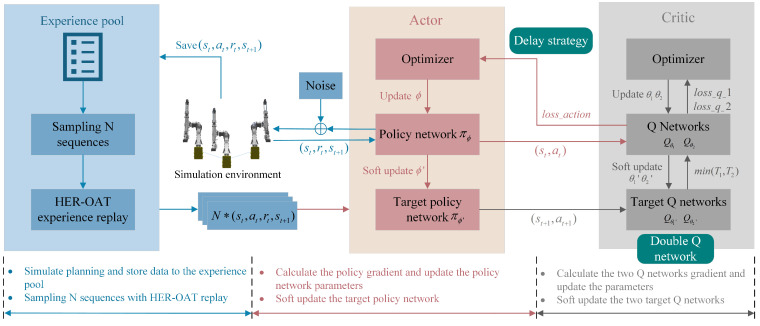
TD3 algorithm framework diagram.

The TD3 algorithm is employed to train a three-arm deep reinforcement learning model for motion planning within the Pytorch framework. TD3 utilizes dual Q-learning with two independent Q networks, selecting the smaller value as the Q value estimate for policy updates to mitigate overestimation bias. A delay strategy is implemented for updating the policy network, where the policy network is updated once after several updates to the critical network, reducing instability caused by value estimation errors. The framework of the TD3 algorithm used in this study is shown in Figure 6.

The state space $S_{t}=\left\{\theta_{main}^{now},\theta_{main}^{goal},\theta_{obs}^{left},\theta_{obs}^{right}\right\}$ consists of $\theta_{main}^{now}$, $\theta_{main}^{goal}$, $\theta_{obs}^{left}$, and $\theta_{obs}^{right}$, with a total of 28 dimensional state variables.

The action space is configured according to the range of motion of each joint of the main robotic arm. To avoid drastic changes in the joint angles, a Tanh activation function is added at the end of the action model so that the output value is limited to the interval from −1 to 1 without adding noise. Meanwhile, to ensure the consistency of the weights between the rotational and translational joints for the subsequent calculations, the unit of the translational joints is set to decimeters.

The determination of superior and inferior paths in DRL model training is directly affected by the reward function $r_{t}\left(a_{t},s_{t}\right)$. In this paper, the reward function consists of three parts, which are the difference reward and penalty of the joint values in two consecutive states $r_{diff}$, the collision penalty $r_{coll}$, and the arrival reward $r_{reach}$, as shown in Equations (8)–(11).

(8) rdiff=((θnow1–7t−θtag1−7)−(θnow1−7t−1−θtag1−7))×k

(9) $$r_{coll}=\left\{\begin{array}{c} -V_{collision},\;if\;collision\\ 0,\;if\;non-collision \end{array}\right.$$

(10) $$r_{reach}=\left\{\begin{array}{c} V_{reach},\;all\left(\theta_{now}^{1-7}-\theta_{tag}^{1-7}\right)<thr\\ 0,\;others \end{array}\right.$$

(11) $$r_{t}\left(a_{t},s_{t}\right)=r_{diff}+r_{coll}+r_{reach}$$

#### 4.2.2. Collision Detection

**Figure 7:**
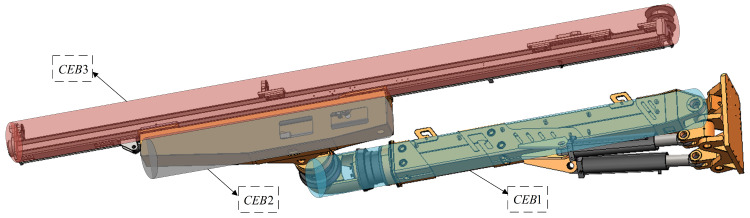
Robotic arm envelope box model.

The single-arm self-collision detection and multi-arm mutual collision detection of the rock drilling robot are transformed into solving the problem of minimum distance between the envelope boxes of spatial cylinders. According to the working conditions of the robotic arm, the collision-prone area is analyzed and divided into three sections for collision detection. These three sections are simplified as cylindrical envelope boxes: the upper arm (CEB1), the lower arm box (CEB2), and the beam (CEB3). Single-arm collisions are most likely to occur between CEB1 and CEB2, as well as between CEB1 and CEB3. Multiple-arm collisions with each other may occur at any two rods, which need to be detected sequentially. The mechanical arm envelope box model diagram is shown in Figure 7.

The detection processes of single-arm collision and multi-arm collision are as follows:

1. Calculate the spatial coordinate position: According to the link coordinate system and the actual size of the robot arm, calculate the spatial coordinate positions P1, P2, Q1, and Q2 of the endpoint values of each cylinder main axis segment.
2. Calculate the closest distance: Calculate the closest distance between two spatial line segments.
3. Collision detection: The closest distance minus the radius of the two cylinders to realize the detection of single-arm collision and multi-arm collision.

The safety status of the robotic arm can be quickly determined by collision detection to provide safety for subsequent motion planning.

#### 4.2.3. HER-OAT Experience Replay Mechanism

**Figure 8:**
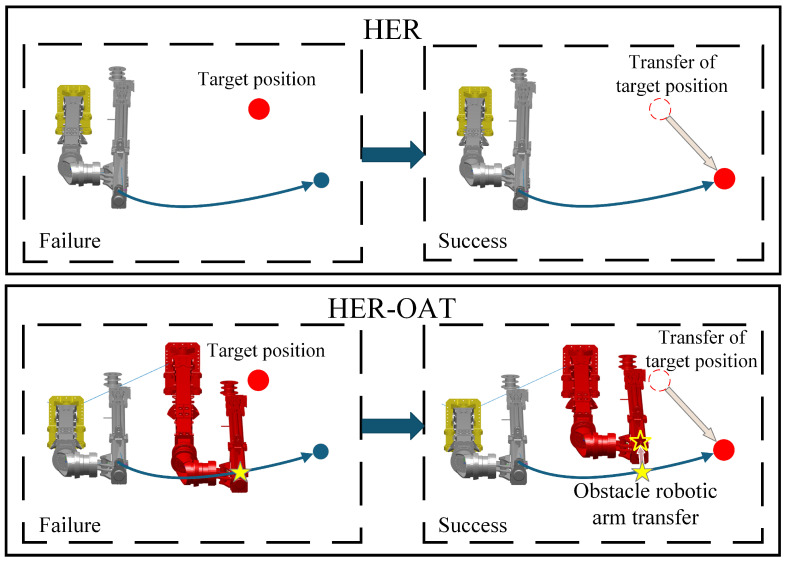
HER-OAT method.

In order to improve the training efficiency, this paper proposes the HER-OAT method to play back the data and convert some of the failed planning into successful planning. The HER method significantly improves the successful planning probability by reselecting the target joint values $\theta_{main}^{goal}$. On this basis, the HER-OAT method carries out a reasonable movement of the $A_{obs}$, thus avoiding the collision between robotic arms to form an effective path. First, based on the collision position data returned from the collision detection, determine whether the collision occurs in the Y or Z direction. Next, using the joint values $\theta_{obs}$$\theta_{main}^{now}$ of both the obstacle and main robotic arms, identify the optimal movement direction for the $A_{obs}$. The Z-axis movement direction is determined by comparing the pitch values of the two robotic arms, while the Y-axis movement direction is determined by comparing their swing values. To ensure path optimization, the $A_{obs}$ undergoes 10 movement cycles, with each cycle adjusting the joint values by ±1°. If collisions persist after completing all cycles, the system is considered to be in a collision state. The HER-OAT method is shown in Figure 8.

The pseudo-code of the TD3 algorithm combined with HER-OAT is shown below (Algorithm 1).
**Algorithm 1:** TD3 (HER-OAT)Use random parameters $\theta_{1}$, $\theta_{2}$, ϕ to initialize the value networks $Q_{\theta_{1}}$, $Q_{\theta_{2}}$ and the policy network $\pi_{\phi }$

Initialize the target networks ${\theta_{1}}^{\prime }\leftarrow \theta_{1}$, ${\theta_{2}}^{\prime }\leftarrow \theta_{2}$, $\phi^{\prime }\leftarrow \phi$
Initialize the replay buffer *B*;
**for** t=1 *to T* **do**

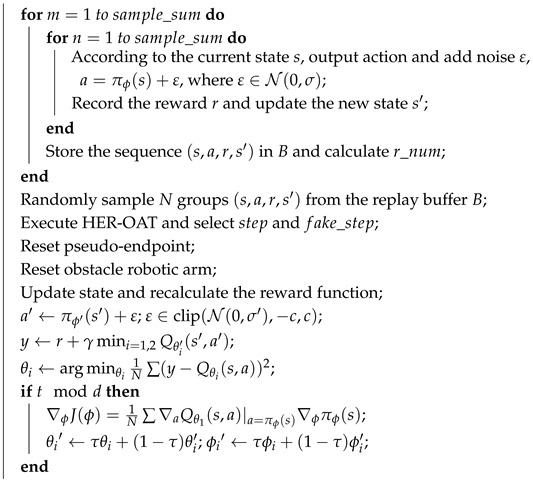

**end**

#### 4.2.4. DRL Model Training Results

**Figure 9:**
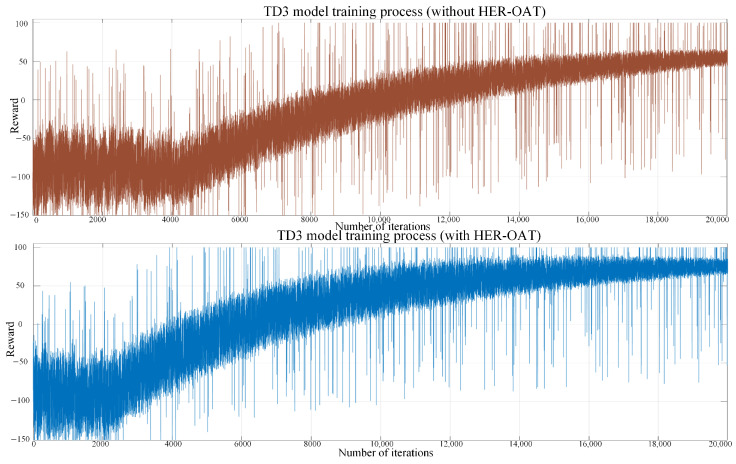
Model training reward curve.

The training reward curves of the TD3 model using the HER-OAT method and not using this method are shown in Figure 9.

The reward of the model using the HER-OAT method stabilizes after 8000 iterations, whereas the model without this method requires 16,000 iterations to approach stability, and the reward does not reach its maximum value. This demonstrates that the HER-OAT method significantly enhances both training efficiency and performance. However, as shown in Figure 9, some instability remains. In the later iterations, collisions may still occur, and the planned path may not be globally optimal.

**Figure 10:**
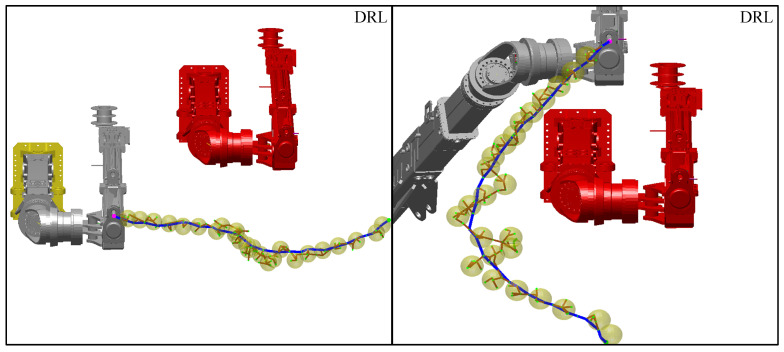
Using DRL position ball sampling alone.

The planning results using the DRL model alone are shown in Figure 10, where the brown curve indicates the optimal path and the blue curve indicates the path resulting from planning.

As shown in Figure 10, when relying on the DRL model alone for motion planning, it is susceptible to environmental noise interference, which makes it difficult to adequately ensure path safety and optimality. Especially in the vicinity of obstacles, the performance of the DRL model is degraded, and it is easy to fall into local fluctuations and be far away from the optimal path. However, in the initial stage of planning and the goal-approaching stage, the DRL model shows excellent performance.

### 4.3. MSS-RRT* Motion Planning Solution

The MSS-RRT* motion planning solution integrates multiple advanced algorithms, adopts the target-corrected dual Jacobi iteration method to achieve accurate inverse solution, leverages the cylindrical envelope box method for efficient collision detection, uses a multi-strategy sampling mechanism to enhance the performance of RRT* path planning, and utilizes the B-spline interpolation method for path smoothing.

**Figure 11:**
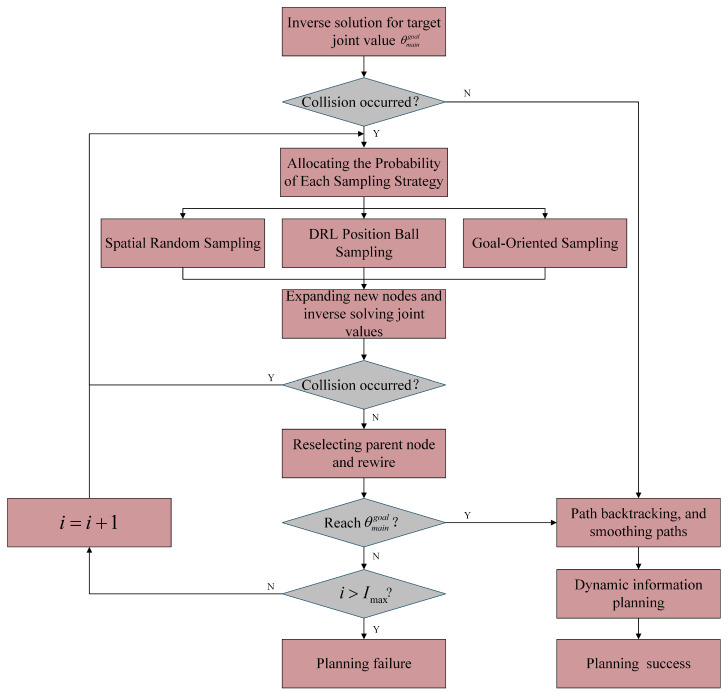
MSS-RRT* motion planning process.

The MSS-RRT* motion planning process is shown in Figure 11, with the following detailed steps:

1. First, the accurate target joint values are calculated using the target-corrected dual Jacobi iteration inverse solution. The intermediate joint values obtained from the two Jacobi iterations are then checked for collisions. If no collision is detected, dynamic trajectory planning is performed directly. If a collision is detected, the MSS-RRT* method is employed for motion planning.
2. Configure the sampling method probabilities based on the current position. DRL position ball sampling constructs the state using the current joint values, target joint values, and obstacle joint values. The state is input into the DRL model to obtain the action, which determines the end position based on the joint value changes. This position is then used as the center of a ball for sampling within the ball. Goal-oriented sampling randomly samples around the target position. Spatial random sampling randomly selects sampling points within the robot arm’s effective space.
3. Collision detection is performed on the new sampling point, which is discarded if a collision occurs and retained if no collision occurs.
4. Perform the reselection of parent nodes and rewiring operations of the MSS-RRT* algorithm.
5. Check if the target joint value is reached. If not, repeat steps 2 and 3. Once the target joint value is reached, perform path backtracking and smoothing, followed by dynamic information planning to complete the path planning task.
6. If the maximum number of iterations is reached and the target joint value has not been achieved, the planning is considered a failure.

The MSS-RRT* algorithm pseudo-code is shown below (Algorithm 2).   
**Algorithm 2:** MSS-RRT***Input**: $V\leftarrow \{x_{init}\}$, E←{∅}, $x_{goal}$, $\theta_{obs}$, $\theta_{main}^{init}$, $\theta_{main}^{goal}$, stepsize, $I_{max}$, Tr **Output**: G=(V,E) 
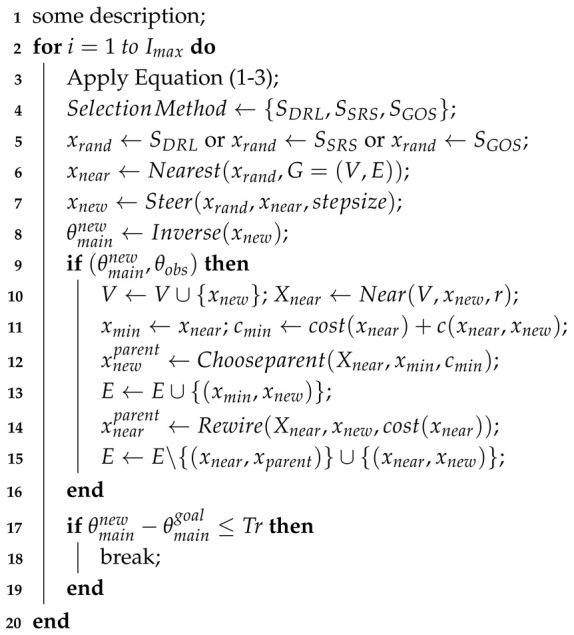


The rough change curve of each joint is obtained by path backtracking, and finally the curve is smoothed by B-spline interpolation method.

## 5. Experiments and Simulations

This section will sequentially present experiments on inverse solution localization accuracy, path planning, and joint smoothing, followed by trajectory planning simulations for ROS systems.

### 5.1. Inverse Solution Localization Accuracy Experiment

**Figure 12:**
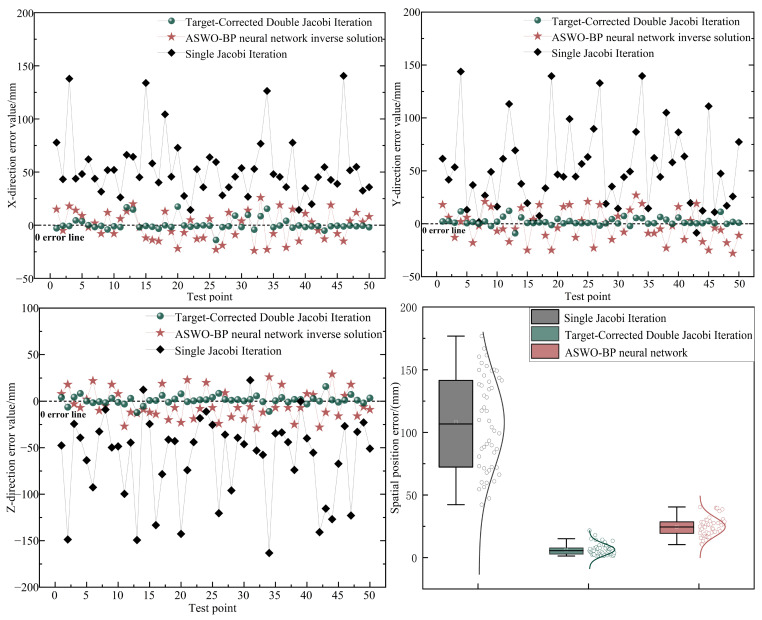
Inverse solution error comparison.

To verify the accuracy of the target-corrected dual Jacobi iteration inverse solution method, 50 sets of inverse solution experiments were conducted. The proposed method is compared with the single Jacobi iteration and the ASWO-BP neural network inverse solution method, where the ASWO-BP neural network establishes a mapping between the actual position of the end effector and the robotic arm joint values, resulting in higher inverse solution accuracy [31]. The experimental results are shown in Figure 12.

The average localization errors in the X, Y, and Z directions for the single Jacobi iteration are 54.43 mm, 53.16 mm, and 60.44 mm. In contrast, for the target-corrected dual Jacobi iteration, the average localization errors in the three directions are 4.56 mm, 3.78 mm, and 4.75 mm, representing improvements of 91.62%, 92.89%, and 92.14%. These errors are also lower than the three-direction average localization errors of the ASWO-BP neural network inverse solution method, which are 11.84 mm, 13.78 mm, and 14.95 mm. From the box plots of the spatial position errors of the three methods, the mean and standard deviation of the three methods are 106.09 ± 37.82 mm, 6.21 ± 3.29 mm, and 24.75 ± 7.55 mm, respectively. Among them, the spatial position error of the method in this paper (6.21 ± 3.29 mm) is significantly lower than that of the other two methods, and the degree of dispersion of the error is smaller. In addition, the method in this paper performs optimally in terms of the maximum value and median value of the error.

### 5.2. Path Planning and Joint Smoothing

First, the planning processes of the four algorithms are visualized through path planning illustrations. Then, based on 30 sets of path planning data, the performance metrics of the four algorithms are compared and analyzed. Finally, the results of joint smoothing and the end-effector trajectory are presented.

#### 5.2.1. Path Planning Illustration

**Figure 13:**
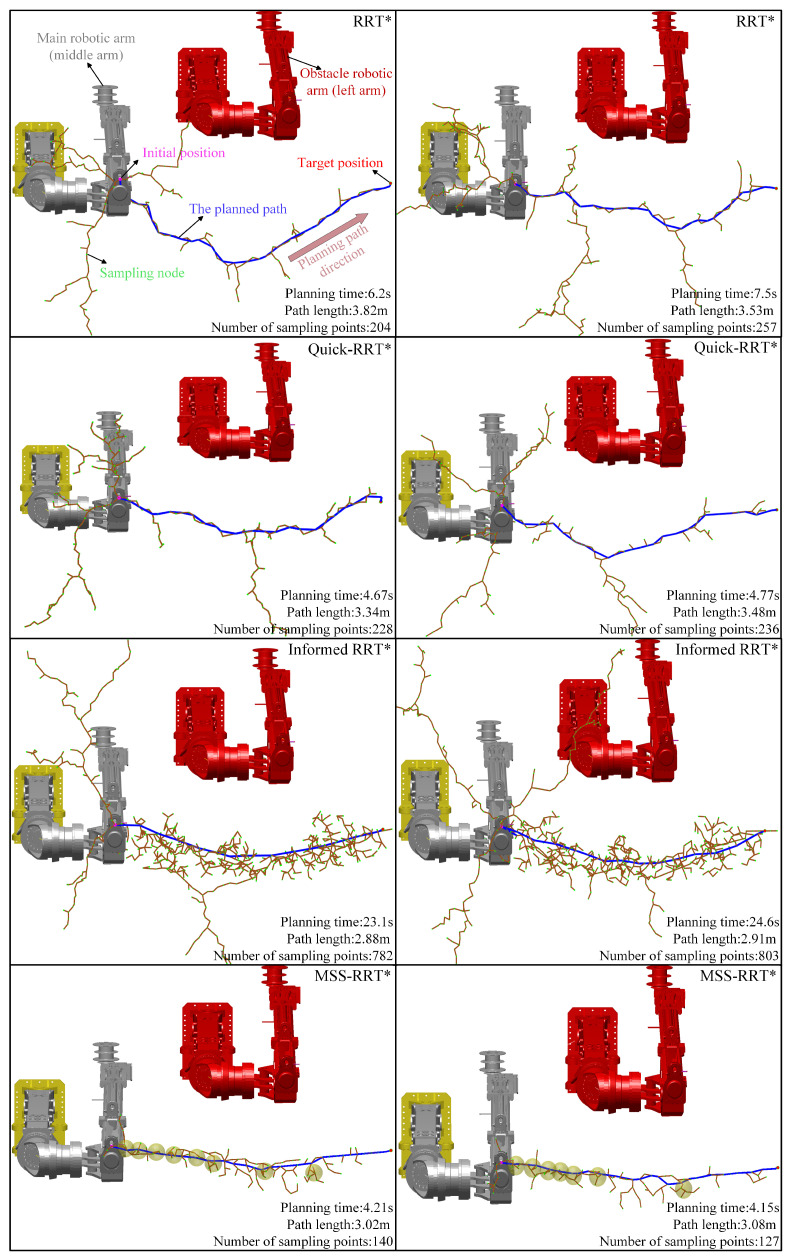
Path planning illustration.

A simulation platform for robotic arm path planning is constructed using MATLAB 2023b software, running on an Intel I7 14650HX CPU. To verify the effectiveness of the MSS-RRT* algorithm in path planning, the RRT* algorithm (the basic algorithm, set to stop when it finds the path), the Quick-RRT* algorithm (by accelerating the optimization to obtain a faster planning time), and the Informed-RRT* algorithm (which produces an asymptotically optimal path) are chosen for comparison. The path planning results of the four algorithms are shown in Figure 13.

The settings for $\theta_{main}^{start}$ and $\theta_{obs}$ are (0°, 0°, 0 mm, 0°, 0°, 0°, 0 mm), and the settings for $\theta_{main}^{goal}$ are (37°, 2°, 933 mm, 0°, −2°, −37°, 309 mm). In the figure, the gray robotic arm represents $A_{main}^{start}$, the red robotic arm represents $A_{obs}$, the purple point denotes the start position, the red point denotes the target position, the green points represent the nodes, the brown lines indicate the node connections, and the blue line represents the feasible path planned by the algorithm.

As shown in Figure 13, the undirected sampling in the initial stage of the RRT* algorithm generates a large number of invalid sampling points. Meanwhile, the limited number of valid sampling points results in more inflection points, causing the path to zigzag. The Quick-RRT* algorithm achieves faster path planning, but the paths are not optimal by accelerating and optimizing the sampling expansion, parent node selection, and rewiring processes. The Informed-RRT* algorithm, after successfully planning the initial path, reduces the generation of some invalid sampling points by sampling within a defined elliptical effective region. While this approach finds a better path, it requires a longer planning time. In contrast, the MSS-RRT* algorithm avoids invalid directional sampling in the first segment using DRL position sphere sampling. In the middle segment, it optimizes the path near obstacles through spatial random sampling, mitigating the failure and collisions associated with DRL position sphere sampling. In the final segment, it quickly reaches the target position through goal-oriented sampling, resulting in an overall path that closely approximates the optimal solution.

#### 5.2.2. Statistical Analysis of 30 Sets of Path Planning

Thirty sets of $\left\{\theta_{main}^{start},\theta_{main}^{goal},\theta_{obs}\right\}$ were randomly selected. Due to the stochastic nature of the sampling-based path planning algorithm, each group was planned 10 times. The length of the robotic arm’s end-effector path, the number of sampling points, the planning time, and the number of failures were recorded. (MSS-RRT*, Quick-RRT*, and RRT* algorithms are considered planning failures if they exceed 500 iterations.)

The four key data sets in Table 4 were statistically analyzed, and *p*-values as well as 95% confidence intervals were calculated to assess statistical significance, practical relevance, and reliability.

1. The comparison of path lengths between the MSS-RRT* and RRT* algorithms shows a mean difference of 1.15 (95% CI [0.96, 1.34], *p* < 0.001), indicating a stable and statistically significant difference between the two groups.
2. The comparison of path lengths between the MSS-RRT* and Informed-RRT* algorithms shows a mean difference of 0.09 (95% CI [−0.08, 0.26], *p* = 0.289), indicating no statistically significant difference between the two groups.
3. The comparison of planning time between the MSS-RRT* and RRT* algorithms shows a mean difference of 2.7 (95% CI [2.35, 3.05], *p* < 0.001), confirming a stable and statistically significant difference between the two groups.
4. The comparison of planning time between the MSS-RRT* and Quick-RRT* algorithms shows a mean difference of 0.3 (95% CI [0.025, 0.485], *p* = 0.031 < 0.05), providing confidence that there is a small and stable difference between the two groups.

As shown in Table 4, in terms of the optimal path, the average path length of MSS-RRT* in 30 sets of experiments is 3.67 m, which is close to Informed-RRT*’s 3.58 m, indicating that MSS-RRT* plans a near-optimal path in only one-fifth of the planning time of Informed-RRT*. In terms of the number of sampling points, the average number of sampling points used for planning by the MSS-RRT* algorithm is 172, which is less than the 267 of the RRT* algorithm, the 258 of the Quick-RRT* algorithm, and the 836 of the Informed-RRT* algorithm. The number of sampling points directly affects the planning time, with the MSS-RRT* algorithm taking an average of 5.2 s, which is slightly lower than the Quick-RRT* algorithm’s 5.5 s and significantly lower than the RRT* algorithm’s 7.9 s. In the 30 sets of experiments, the MSS-RRT* algorithm had only one failed planning, while the Quick-RRT* algorithm and the RRT* algorithm had three and six, respectively.

#### 5.2.3. Joint Smoothing and End Trajectory

**Figure 14:**
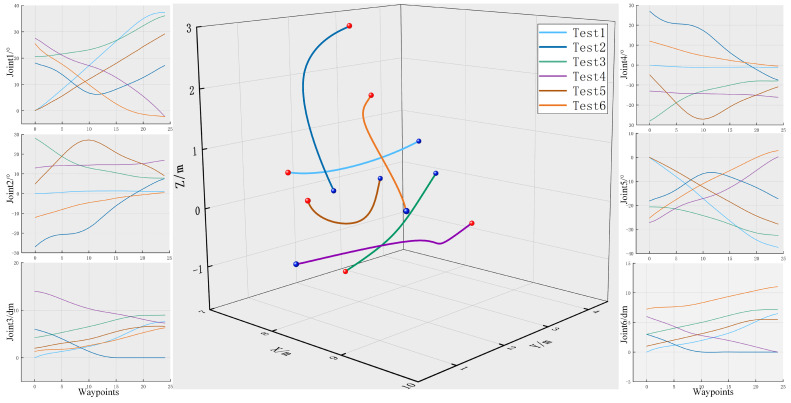
Joint variation and end-effector trajectory curves.

Six path planning experiments were conducted to record the change data of each joint of the robot arm (the change amplitude of the robot arm beam flip joint is small, so it is ignored here). After smoothing by B-spline interpolation method, the change curves of the six joints and the change curve of the spatial position of the end of the robot arm in the base coordinate system are shown in Figure 14. The red point in the figure is the starting point of the path, and the blue point is the end point of the path.

As shown in Figure 14, there are no obvious inflection points in the change curves of each joint, indicating that the B-spline interpolation method effectively ensures the smoothness of joint movement. The smooth change in the joint makes the end running trajectory smooth as well, effectively avoiding the shaking of the robot arm and ensuring the smooth operation of the robot arm.

### 5.3. ROS System Trajectory Planning Simulation

Based on the rock drilling robot arm URDF file, a three-arm simulation model of the rock drilling robot is constructed to accurately restore the geometric dimensions and physical properties of the experimental object. The discrete joint position information is input into the ROS system for interpolation processing to calculate the speed and acceleration information. The TimeParameterization module in MoveIt is used to time-parameterize the joint trajectory and optimize the time allocation of the trajectory to ensure that the movement of the robot arm is smooth and executable while meeting the speed and acceleration constraints. Finally, the motion trajectory of the robot arm is visualized in Rviz.

**Figure 15:**
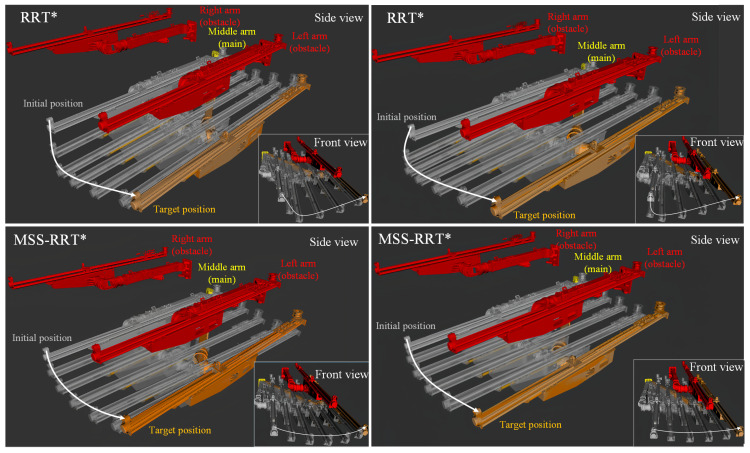
Trajectory planning simulation.

In the same state, multiple experiments were conducted using the MSS-RRT* and RRT* algorithms to verify the stability of each method, as shown in Figure 15. The joint settings are the same as those in the path planning experiment. The red robot arm in the figure is $A_{obs}$, the yellow robot arm is $A_{main}^{goal}$, the gray robot arm represents the planning process of $A_{main}$ from the initial pose to the target pose, and the white curve represents the terminal running trajectory of $A_{main}$. In order to clearly observe the running status of the robot arm, the planning situation is observed from the side view and the front view, respectively.

As shown in Figure 15, the MSS-RRT* algorithm is significantly better than the RRT* algorithm in terms of the length of the end trajectory and the joint inflection points. In terms of stability, the RRT* algorithm produces paths with significant variation, resulting in longer paths in some cases. In contrast, the MSS-RRT* algorithm generates more consistent paths, effectively avoiding obstacles and staying closer to the optimal trajectory.

## 6. Conclusions

The dual Jacobi iteration method based on target correction improves spatial localization accuracy by 94.15% compared to the single Jacobi iteration method, and by 74.95% compared to the ASWO-BP neural network inverse solution method. The proposed HER-OAT method significantly enhances DRL model training efficiency. The multi-strategy sampling mechanism of the MSS-RRT* algorithm strategically applies three sampling methods at different stages of motion planning, effectively reducing the generation of invalid sampling points and guiding the paths toward optimal solutions in a short time. The experimental results demonstrate that MSS-RRT* plans a near-optimal path in only 20.71% of the planning time required by Informed-RRT*. With similar planning times, MSS-RRT* reduces the path length by 21.58% compared to Quick-RRT* while offering superior security and stability.

The method proposed in this paper provides an innovative solution for high-precision motion planning of multi-manipulator arm systems and shows significant application potential in complex task scenarios. However, this study still has the following limitation: in dynamic obstacle environments, the real-time performance of robotic arm motion planning is limited by the computational efficiency of the current algorithm, which has difficulty meeting the real-time response requirements of highly dynamic scenes. Future research should focus on breaking through the real-time computational bottleneck of dynamic motion planning, which can be explored in depth in the following directions:

1. Accelerating the sampling process with GPU parallel computing architecture to improve planning efficiency.
2. Establishing a real-time dynamic response mechanism based on prediction-correction, enhancing system real-time performance through feedforward compensation and feedback adjustment.
3. Developing a multimodal obstacle motion state estimation system by integrating vision and depth sensor information.

## Figures and Tables

**Table 1:** DH parameters table.

i	$\theta_{i}/{}^{\circ}$	$d_{i}/$mm	$\alpha_{i}/{}^{\circ}$	$a_{i}/$mm	Joint Range
1	$\theta_{1}$ (0)	0	−90	200	$\theta_{1}$ (−40,40)
2	$\theta_{2}$ (90)	0	90	−35	$\theta_{2}$ (−50,40)
3	90	$d_{3}$ (4297)	0	0	$d_{3}$ (0,2200)
4	$\theta_{4}$ (90)	0	90	0	$\theta_{4}$ (−180,180)
5	$\theta_{5}$ (90)	650	90	80	$\theta_{5}$ (−130,130)
6	$\theta_{6}$ (90)	679.5	90	241	$\theta_{6}$ (−12,58)
7	0	$d_{7}$ (3058)	0	0	$d_{7}$ (0,1600)

**Table 2:** Symbol reference table.

Symbol	Definition	Symbol	Definition
$\theta_{now}^{1-7}$	Current initial joint values	$P_{one}^{tgt}$	Target end-effector position
$T_{one}^{tgt}$	Initial target pose matrix	$P_{one}^{error}$	Error value between $P_{one}^{tgt}$ and $P_{one}^{act}$
$\theta_{one}^{1-7}$	Joint values obtained from the first inverse kinematics solution	$P_{two}^{tgt}$	Corrected target position
$P_{one}^{theo}$	Theoretical end-effector position corresponding to $\theta_{one}^{1-7}$	$\theta_{upd}^{1-7}$	Joint values updated after the first inverse kinematics solution
$P_{one}^{comp}$	End-effector error compensation corresponding to $\theta_{one}^{1-7}$	$T_{two}^{tgt}$	Updated target pose matrix
$P_{one}^{act}$	Actual end-effector position corresponding to $\theta_{one}^{1-7}$	$\theta_{two}^{1-7}$	Precise joint values obtained from the second inverse kinematics solution

**Table 3:** Symbol reference table.

Symbol	Definition	Symbol	Definition
$A_{main}$	Main robotic arm (middle arm)	$P_{SRS}$	Probability of spatial random sampling
$A_{main}^{start}$	Initial state of $A_{main}$	$P_{GOS}$	Probability of goal-oriented sampling
$A_{main}^{now}$	Current state of $A_{main}$	$d_{now}^{goal}$	Euclidean distance between the end effectors of $A_{main}^{now}$ and $A_{main}^{goal}$
$A_{main}^{goal}$	Goal state of $A_{main}$	$d_{thresh}^{goal}$	Euclidean distance between the end effectors of $A_{main}$ and $A_{main}^{goal}$ at critical state
$A_{obs}$	Obstacle robotic arm (left arm and right arm)	$\theta_{main}^{start}$	Initial joint values of $A_{main}$
$D_{now}^{obs}$	Minimum distance between $A_{main}^{now}$ and $A_{obs}$	$\theta_{main}^{now}$	Current joint values of $A_{main}$
$D_{start}^{obs}$	Minimum distance between $A_{main}^{start}$ and $A_{obs}$	$\theta_{main}^{goal}$	Goal joint values of $A_{main}$
$D_{thresh}$	Critical minimum distance	$\theta_{obs}^{left}$	Joint values of the obstacle robotic arm (left)
$P_{DRL}$	Probability of DRL position ball sampling	$\theta_{obs}^{right}$	Joint values of the obstacle robotic arm (right)

**Table 4:** Thirty sets of planning data statistics.

Group	Algorithm	End Path Length/m	Number of Sampling Points	Planning Time/s	Number of Failures
Group 1	MSS-RRT*	2.97–3.21	122–150	3.9–4.1	0
	RRT*	3.43–4.36	204–285	6.1–9.2	0
	Informed-RRT*	2.88–3.04	752–906	22.6–26.5	-
	Quick-RRT*	3.34–4.45	216–281	4.5–4.9	0
Group 2	MSS-RRT*	3.89–4.15	184–202	5.5–6.1	0
	RRT*	4.37–5.72	221–315	6.7–9.5	1
	Informed-RRT*	3.81–4.06	763–943	23.1–28.3	-
	Quick-RRT*	4.35–5.41	219–288	5.6–6.3	0
⋮	⋮	⋮	⋮	⋮	⋮
Group 30	MSS-RRT*	3.34–3.57	161–190	5–5.8	0
	RRT*	3.86–4.63	203–261	6.1–7.9	0
	Informed-RRT*	3.22–3.39	720–857	22.3–25.7	-
	Quick-RRT*	3.64–4.68	199–271	5.2–6	0
Total average	MSS-RRT*	3.67	172	5.2	1 (cumulative)
	RRT*	4.82	264	7.9	6 (cumulative)
	Informed-RRT*	3.58	836	25.1	-
	Quick-RRT*	4.68	258	5.5	3 (cumulative)

## Data Availability

The data used to support the findings of this study are available from the corresponding author upon request. The data are not publicly available due to privacy.

## Abbreviations

The following abbreviations are used in this manuscript:

MSS-RRT*	Multi-strategy sampling RRT*
DRL	Deep reinforcement learning
TD3	Twin delayed deep deterministic policy gradient
HER-OAT	Hindsight experience replay-obstacle arm transfer
DRL-PBS	DRL position ball sampling
SRS	Spatial random sampling
GOS	Goal-oriented sampling
